# Weight-loss associated DNA methylation patterns: targetable biomarkers and pathway insights

**DOI:** 10.1186/s13104-025-07324-x

**Published:** 2025-07-22

**Authors:** Longjin Zeng, Xu Chen, Longyao Zhang, Yaxian Qi, Lingchen Li, Chenrui Yin, Jianguo Sun

**Affiliations:** 1 Department of Basic Medicine, Army Medical University, Chongqing, 400038 China; 2 Department of Medical Affairs, Xinqiao Hospital, Army Medical University, Chongqing, 400037 China; 3 Cancer Institute, Xinqiao Hospital, Army Medical University, Chongqing, 400037 China

**Keywords:** Bariatric surgery, Skeletal muscle, CPTAC, EPIC v1.0 array, Consistence evaluation

## Abstract

Weight loss is a firmly established negative survival factor for individuals with cancer, yet effective biomarkers and targeted therapies remain elusive. In this study, we collected skeletal muscle, noncancerous, and cancerous tissues using the Illumina EPIC array to identify conserved DNA methylation probes associated with weight loss following bariatric surgery. Next, the consistency of the probes is evaluated and then the probes are integrated into a generalizable pathway enrichment score. Our results emphasize the gene-centered design, identifying *KCNB1*, *PEAK1*, *SCG5*, and *TNIK* as key targets of DNA methylation, as confirmed by mouse phenotype data and druggability resources. Moreover, an illustrative test of protein abundance in cell lines is conducted. Utilizing the Clinical Proteomic Tumor Analysis Consortium data, a positive correlation is established between the chromosomal instability scores and our generated score in tumor tissues. In addition, considering these correlation findings, the presence of identifiable methylation blocks in the co-occurring gain samples. Our findings also suggest that upstream molecular drivers may influence this pathway enrichment score, potentially leading to dysregulated methylation associated with weight loss. In summary, DNA methylation analysis not only identifies functional targets but also uncovers new gene-disease connections.

**Supplementary Information:**

The online version contains supplementary material available at 10.1186/s13104-025-07324-x.

## Introduction

Cancer cachexia is a multifactorial syndrome characterized by significant and rapid weight loss, which adversely affects patient prognosis. Current research primarily focuses on secreted molecules, but effective biomarkers and therapeutic targets remain elusive [1, 2]. Disease phenotypes can be influenced by environmentally shaped epigenetic memory. Epigenetics, particularly DNA methylation (DNAm), which is the most extensively studied mechanism, refers to processes occurring outside of genetic changes [3].

The reprogramming of skeletal muscle, which may be linked to cellular differentiation, is subject to epigenetic modifications [4]. Metabolic surgery is the most effective long-term method for weight reduction, and recent studies with paired designs have detected widespread DNAm changes in skeletal muscle samples, regardless of sex [5, 6]. Conserved methylation patterns may reveal their interactions with genetic alterations. A correlation study showed localized DNAm associated with somatic copy number alterations (SCNAs) in TCGA data [7]. Meanwhile, the states of amplification regions can be predicted by DNAm patterns [8]. Indeed, the relevance of neighboring DNAm decreases when the distance exceeds 1 kilobase, highlighting the need to sample co-methylated blocks in genome-wide arrays with sufficient density [9].

The Illumina EPIC array increases its probe count by 350,000 over the previous 450K version, but the β values of individual probes often violate the assumptions of linear regression [10, 11]. Alternative approaches include weighted enrichment scoring and region-based analysis. Well-characterized regions, usually located in CpG islands, are involved in transcriptional repression. However, CpG islands may not be reliable on the EPIC platform [12]. This is supported by evidence that probes targeting non-island genomic locations can distinguish biological states, such as microsatellite instability [13]. Overall, the values themselves need to be evaluated for consistency, which may affect the reproducibility of the probes.

The Clinical Proteomic Tumor Analysis Consortium (CPTAC) integrates the EPIC platform with genomics, focusing on gene-centered analysis [14]. Epistatic oncogenic drivers and non-genetic components together shape tumor evolution. Additionally, DNAm profiles can reflect cellular origins. This manuscript explores the coupling of drivers with methylation patterns in weight loss.

## Materials and methods

### Processed datasets

Public datasets were collected and analyzed independently. We utilized DNAm arrays from the Illumina EPIC platform, including GSE135063, GSE272137, GSE213478, GSE151407, and CPTAC [5, 6, 14–16]. Instead of processing the original signal intensities, we directly downloaded the β ratio matrix and examined the processing pipelines. The main objectives were: (1) to obtain consensus features before and after metabolic surgery; (2) to evaluate features and generate weights; and (3) to apply generalized signatures to cancer.

The training data included paired samples from GSE135063 (*n* = 32) and GSE272137 (*n* = 26), encompassing both genders and collected before and after bariatric surgery. Note that the samples in this study remained obese at 52 weeks postoperatively and were all of white European descent. Further probe evaluation was conducted using GSE213478 (*n* = 650) and GSE151407 (*n* = 6). We excluded genital organs, retaining breast, colon, kidney, lung, skeletal muscle, and whole blood samples in GSE213478. For GSE151407, six samples including control and duplicate were used.

Quantification was performed using the β value, which ranges between 0 and 1. It was calculated as “M/(U + M),” where M represents the methylated signal, and U represents the unmethylated signal. Samples with more than 80% missing data will be excluded, and missing values will be imputed using the nearest neighbors method. We utilized the R package **CHAMP** (version 2.28.0) for probe annotation and subsequent analysis [17]. Probe categories included “1st Exon,” “TSS200,” “TSS1500,” “body,” “UTR,” or “IGR.” Meanwhile, probes could also be annotated as “island,” “shore,” “shelf,” or “open sea” using the UCSC browser as a reference. For differential analysis of DNAm sites, we utilized M-value transformation, defined as the log2 ratio of methylated probe intensity to unmethylated probe intensity:

$$\:\left(\text{M}=\text{l}\text{o}\text{g}2\frac{{\upbeta\:}}{1-{\upbeta\:}}\right)$$

### CPTAC source files and usage

To meet the need for DNAm data in adjacent normal tissues, five items including lung squamous carcinoma (LSCC), lung adenocarcinoma (LUAD), head and neck squamous carcinoma (HNSCC), clear cell renal cell carcinoma (ccRCC) and pancreatic ductal adenocarcinoma (PDAC) were considered in this study. The following files were downloaded from CPTAC Pan-Cancer Data (https://pdc.cancer.gov/pdc/cptac-pancancer): “methylation array - Methylation_WashU_v1”, “mutation data - Mutation_BCM_v1”, “CNV data - CNV_WGS_WashU_v1”, “clinical and other characteristics - Clinical_meta_data_v1”.

In terms of clinical data, our focus was on body mass index (BMI), age, tumor mutation burden (TMB), and chromosome instability (CIN) score. The CIN score encapsulates the aggregate impact of copy number variations across all chromosomes [18]. Chromosome calling fragments using genome-wide data from the BIC-seq2 pipeline, with the final total score summed across chromosomes. Additionally, mutations were somatic variants, and could be depicted using the file “Mutation_Broad_WashU_union_v1.”

### Consulted mouse phenotypic and druggable genome resources

The downstream analysis of DNAm probes was gene-centric, involving a total of 600s coding genes. We searched for one-to-one human homologs in mouse, specifically designating the C57BL strain. For mouse knockout phenotypes, we focused on the “growth/size/body region phenotype” (MP:0005378) and “adipose tissue phenotype” (MP:0005375) semantics [19]. As previously described, dysregulation of skeletal muscle and adipose tissues may lead to cancer cachexia [1]. Parameters measured included “Body length,” “Fat/Body weight,” “Fat mass,” “Lean mass,” “Lean/Body weight,” and “Weight.” Genes that showed discordant phenotypes, such as increased fat but decreased lean body mass, were excluded. For this, we emphasize that it is a simplified screening, given that opposite effects can occur in different tissues. Only concordant genes were considered, and the same criteria were applied to the probe filter. Druggability was referenced by Jiang et al., who reported that the majority of recurrent mutations had been targeted by small-molecules or approved drugs [20].

### Correlation and consistency evaluation

Intra-class correlation coefficients were used to reflect the clustering of variables within groups. For two variables in the same group, inter-class correlation was applied, while consistency for univariate data was measured by the change rate. The intra-class correlation was calculated through the random-effects analysis of variance, as described by Cao et al. [21]. Spearman’s inter-class correlation is more applicable to nonnormal data [22]. Consistency assumes that the control and replicate values are identical. The change rate was calculated as the ratio of the absolute difference to the base value, which was derived from the control or replicate. The formula is as follows:

$$\:\text{c}\text{h}\text{a}\text{n}\text{g}\text{e}\:\text{r}\text{a}\text{t}\text{e}=\left(\frac{\left|{{\upbeta\:}}_{\text{c}\text{o}\text{n}\text{t}\text{r}\text{o}\text{l}}-{{\upbeta\:}}_{\text{r}\text{e}\text{p}\text{l}\text{i}\text{c}\text{a}\text{t}\text{e}}\right|}{\text{b}\text{ase\:}\text{v}\text{alue}}\right)\times\:100\text{\%}$$

The criterion for DNAm probe filtration was an intraclass correlation coefficient exceeding 0.4 within Genotype-Tissue Expression (GTEx) samples. GTEx data included both male and female tissues, and the candidate probes were also affected by male/female factors. Next, we used replicates to assess consistency, finding fewer than one hundred probes that changed by more than 100%, primarily those with low β values. In summary, the evaluation results indicate that the candidate probes are reproducible but require further refinement.

### Generalized linear regression

Determination of individual probes may be difficult in some tissues, so it is more robust to employ pathway enrichment. Although training and test set crossovers are commonly used in machine learning, the real situation exists where the weights and the purpose of the training are not well defined. In this study, we assume equal weighting across datasets and require that the scores fall within a specific range. The enrichment score is determined by the individual’s weights and β value with the following formula:

$$\:\text{S}\text{c}\text{o}\text{r}\text{e}={w}_{1}{{\upbeta\:}}_{i1}+\dots\:+{w}_{k}{{\upbeta\:}}_{ik}$$

The generalized elastic net includes L1-norm and L2-norm penalties to compute weights. For each DNAm probes were employed via R package **gelnet** (version 1.2.1) [23]. The package relied on graph laplacian, which assigns similar but not same weights to the given genes, and may help to compress the quantiles (i.e. preferring L2-norm penalties). In contrast to this, penalized likelihood methods (e.g., LASSO) make the coefficients inflated, according to our calculations.

### Differential analysis

Differential DNAm probe analysis was performed using the champ.DMP function, with statistical testing conducted via eBayes. Specifically, the log fold change for DNAm probes was set at 0.1. Additionally, a nonparametric Wilcoxon test was used to identify mutations affecting the scores; mutations were grouped into binary variables, i.e., mutant and wild types. Non-synonymous mutations were classified as mutant types.

### Co-methylated blocks on chromosome 19

This exploratory analysis focused on co-methylated blocks. The R package **EnMCB** (version 1.16.0) was adapted for use on the EPIC platform (**Supplementary file**), utilizing the original manufacturer’s annotated version of ilm10b4.hg19 [24]. Two moderately correlated DNAm probes, which were adjacent, were retained, and could ultimately be merged into a single boundary. The modified “IdentifyMCB_parallel” function parameters were set to (method = c(“spearman”), CorrelationThreshold = 0.4 or 0.6, PositionGap = 1000, platform = “Illumina Methylation EPIC”). Also, the above human genome used the hg19 version.

The example provided here pertains to CPTAC data, where samples were identified as co-occurring gain genes within the 19q12-13.12 region (chr19:28493000–37754420) [25]. A sample was amplified if all genes coding for the region met the conditions, i.e., a log2 copy ratio greater than 0.2. Conversely, samples in the ccRCC cohort did not satisfy the calling conditions. Also, gain genes were best excluded from the list of deletions.

### Cell culture and SCG5 assay

H292, A549, and H226 cell lines were cultured in 1640 medium with 10% serum, while H520 was in DMEM with 10% serum. After 48 h for H292 and A549, and 72 h for H226 and H520, supernatants were collected, centrifuged at 2000 rpm for 20 min, and the clear supernatant was saved as samples.

To evaluate the protein level of SCG5, the enzyme linked immunosorbent assay (ID: E9861h) from EIAab company was used for the experiments. Reagent and sample equilibration, preparation of reagent A, B and washing work solution. Add standards or samples to 96-well plate with preincubated primary antibody, make replicates. Incubate with plate sealer at 37 °C for 2 h. Discard liquid, add reagent A, incubate at 37 °C for 1 h. Wash with working solution, pat dry, add reagent B, incubate at 37 °C for 1 h. Discard liquid, wash plate. Add reaction solution, incubate at 37 °C for 10–20 min protected from light. The final absorbance at 450 nm was measured within 15 min after adding the termination solution. Experiments were conducted in triplicate, and recorded in mean values ± SEM.

### Statistical information

P-values were two-sided, and multiple corrections were applied using the Benjamini & Hochberg method. Values less than 0.05 were considered significant. Heatmaps were generated using the R package pheatmap, and all analyses were conducted using R software 4.2.2 version.

## Results

### Gene-centered design in weight loss-induced DNA methylation profiles

**Fig. 1:**
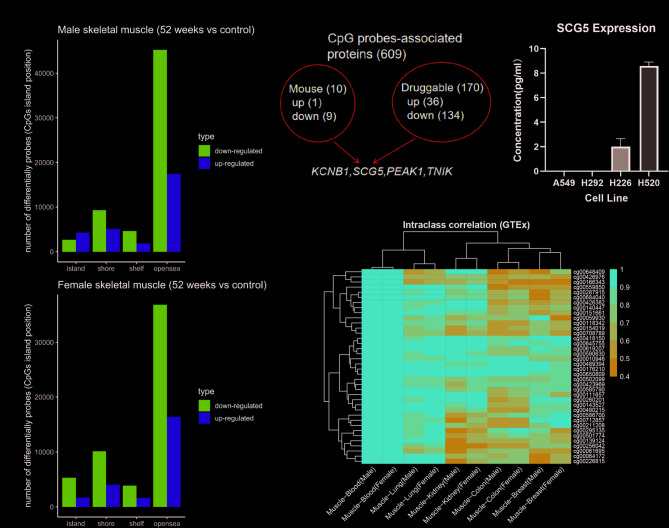
Comprehensive analysis of DNAm probes and corresponding proteins before and after bariatric surgery (A) Different DNAm probes based on their association with CpG islands, grouped by obesity rather than surgery, and at 52 weeks post-surgery (top: male; bottom: female). (B) Schematic showing proteins corresponding to identified DNAm probes, with data sourced from the International Mouse Phenotyping Consortium and the Cancer Druggable Gene Atlas portals. The candidate targets KCNB1, SCG5, PEAK1, and TNIK were validated through mouse knockout experiments and listed in druggable databases. (C) Quantification of SCG5 in four lung cancer cell lines through the enzyme linked immunosorbent assay. Our results showed that all values were roughly below 10 pg/ml, with the highest expression in the H520 cell line. (D) Heatmaps displaying the intra-class correlation of filtered DNAm probes upregulated in the weight-loss group (horizontal axis: paired tissues in the GTEx dataset centered on skeletal muscle; vertical axis: probe names; correlation index range: 0.4-1)

Clinical factors significantly influence DNAm comparisons. In the clinical trial NCT01477957, the two main grouping variables were the presence or absence of type-2 diabetes and gender [5, 6]. We found that DNAm changes in individuals without type-2 diabetes were conserved, with an approximate Jaccard index of 0.26. Among these changes, males showed slightly more alterations than females, with the majority of these changes occurring in the opensea region (Fig. 1A). Consistent with previous research, the majority of probes showed hypermethylation prior to surgery [5, 6, 26]. Note that our grouping setting is “52 weeks after surgery” compared to “before metabolic surgery”, because we are focusing on weight loss.

Gene-centeredness was the primary design for identifying disease targets in this manuscript, with mouse phenotypes and druggability considered. We selected common probes between male and female groups, and then removed genes targeted by up- and down-regulated simultaneous probes (Table S1). Of the 609 coding genes, 10 were supported by mouse experiments, and 170 had small-molecule compounds or clinical drugs for targeting. As another category, secreted or surface proteins have drug potential but require quantification of protein abundance. High priority DNAm mediated targets included *KCNB1*, *PEAK1*, *SCG5*, and *TNIK*, with SCG5 further validated in four lung cancer cell lines (Fig. 1B, C). Our results suggest that in spite of the fact that the abundance of SCG5 can be effectively quantified in tissues, further selection of cell lines suitable for targeting SCG5 is needed [27].

Distinct from the dispersive semantic analysis, our results are based on high throughput data. Using subcutaneous and omental adipose samples, genes such as *CPT1B*, *DPP4*, *FTO*, *HIF3A*, *HOXA4*, *IFFO1*, *INSR*, *IRS1*, *PTPRN2*, *PRDM16*, *PROX1*, *RBMS1*, *SHANK2*, *SMAD3*, *TNXB*, *ZFPM1*, *PCSK6* and *PHACTR1* were differently methylated in post-surgery [26]. Interestingly, we found that the convergent alterations could be found in both skeletal muscle and adipose tissues, although most genes correspond to a single probe.

The regulation of weight loss by DNAm patterns remains unclear; thus, we focused on upregulated DNAm probes in the post-surgical group. Intraclass correlation and the change rate were considered, respectively. Based on an intraclass correlation index of more than 0.4, 38 of the 187 upregulated probes were passed using the GTEx DNAm dataset, suggesting that the screened probes may be similarly hypermethylated or hypomethylated across tissues [15]. Our results showed that the index cluster was influenced by tissue rather than gender (Fig. 1D).

Probe values were tissue related, some genes, such as *DUSP22*, may not be convincingly measured in skeletal muscle. Using six samples, including controls and replicates from GSE151407, we identified the top ten probes with the highest change rate associated with *DUSP22* [16]. Among these probes, cg17876578 was considered a diagnostic biomarker [28].

In other words, clinically meaningful probes do not guarantee that reliable measurements will be made in all tissues. Despite the screened probes have considered gender and tissue differences, the change rate was still as high as 11% (Table S2). Hence, pathway enrichment scores are more robust than individual probes. In this study, training through skeletal muscle data and subsequent testing in the external cohort GSE151407 revealed that higher weights correspond to higher signals, enabling cross-tissue testing.

### Clinical markers, DNAm score, and molecular drivers in the CPTAC cohorts

CPTAC collects clinical information that is valuable for establishing phenotypic associations through omics data. BMI, a common measure of human obesity, typically falls within a normal range of 18 to 25. However, in the CPTAC data, raw BMI values did not correlate with age, TMB, or CIN in CPTAC data (Figure S1). A previous study also reported the challenge of establishing relationships between DNAm and BMI [29]. Instead, we aimed to identify potential targets related to weight loss. Pathway enrichment scores were more likely to adhere to the normal distribution, which is suitable for linear correlation analysis. As a supplement, the weights of the probes used in this analysis are detailed in Table S2.

We calculated correlations between the pathway enrichment score and variables such as BMI, age, TMB, and CIN values. The CIN value was moderately positively associated with both TMB and the score we generated. However, our results did not support the association of scores with BMI, age, or TMB (Figure S2). Consequently, we examined the association between CIN and weight loss scores.

**Fig. 2:**
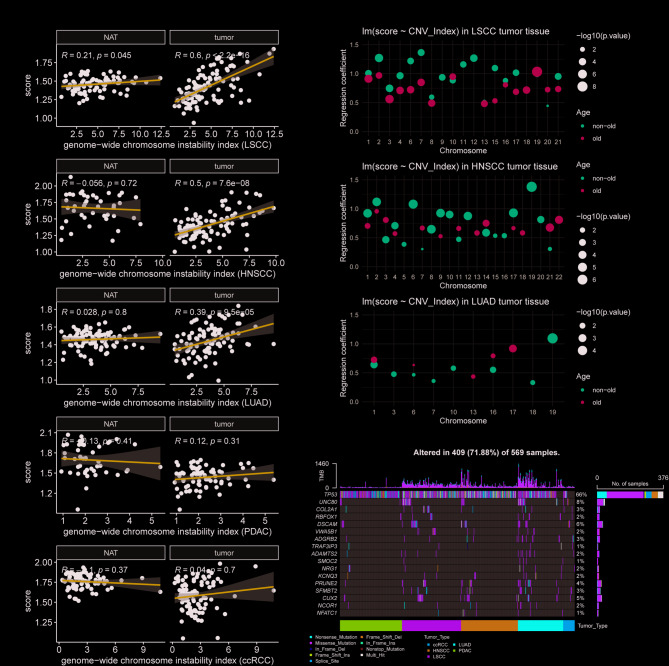
Linkage of DNAm scores to genetics in CPTAC data (A) Inter-class correlation analysis between DNAm pathway enrichment and chromosome instability scores in five CPTAC cohorts, with samples divided into adjacent normal and tumor tissues. (B) Simple linear regression modeling the effects of chromosome instability on DNAm pathway enrichment scores. A moderate global correlation was observed in LSCC, HNSCC, and LUAD tumor tissues, which served as a basis for regression modeling. Only the p-values and their corresponding regression coefficients for significant events were shown here (subgrouping by age over 65 and under 65 years). (C) Overall mutation profiles of five tumor tissue types from CPTAC, focusing on mutations present in more than 3% of each cohort that impact the DNAm pathway enrichment score. All selected mutations, except TP53, were involved in the methylated-mutated two-hit mechanism

Weight loss score could be generated in both tumor and adjacent normal tissues, with LSCC, HNSCC, and LUAD presenting ideal correlations in tumor tissues (Fig. 2A). Assuming that total CIN was divided into individual chromosomes, with age used as a grouping variable. The results indicated that ignoring cohorts with lower correlations, including PDAC and ccRCC, would help improve the modeling performance of simple linear regression (Table S3). Meanwhile, the regression coefficients were relatively flat in the elderly, which may be related to pre-existing skeletal muscle degeneration [2]. We then chose chromosome 19 as an example because of its higher regression coefficients and DNAm signals (Fig. 2B, Table S3). After attempting to vary the correlation coefficients and considering at least five probes in a single block, co-methylated blocks could be preserved in these amplified samples across different datasets, analogous to focal-level events discovery (Table S4). In addition, the observation of comparisons that yield higher correlation thresholds may help to differentiate selected effects by setting up a set of equal numbers of samples.

Finally, interactions of mutations with DNAm were revealed. Pathway scores were tested with mutations as a binary variable, including mutations with rates greater than 3% in each tumor type. There were 246 mutational genes identified in LSCC, LUAD, HNSCC, PDAC, and ccRCC (**Table S5**). Among them, *ADAMTS2*, *ADGRB2*, *COL2A1*, *CUX2*, *DSCAM*, *KCNQ3*, *NCOR1*, *NFATC1*, *NRG1*, *PRUNE2*, *RBFOX1*, *SFMBT2*, *SMOC2*, *TRAF3IP3*, *UNC80* and *VWA5B1* were identified as two-hit genes, affected by both mutation and DNAm (Fig. 2C). Interestingly, in PDAC and ccRCC, the generated score showed an opposite trend about *TP53* mutations. Additionally, scoring identified global methylation drivers, such as *TET2* in LSCC and *KDM5C* in ccRCC.

## Discussion

This study explores the intricate relationships between weight-loss, DNA methylation, and genetics, shedding light on how these characteristics interplay in health and disease. Extending skeletal muscle memory to cancer may facilitate the discovery of targets.

Our study is based on a conserved DNAm profile, with the selection of features considering mixed factors such as gender and tissue. Single DNAm probe and BMI values have low efficiency in linear analyses in the cancer context. Instead, pathway scoring is more theoretically robust and has a higher likelihood of conforming to a normal distribution. This elastic net method relies on the chosen features rather than the training or test data and is particularly applicable when the outcome variable is unclear. Importantly, our approach is general and can be applied to other phenotypes, such as neurodevelopment, as described (Figure S3).

Data-driven analyses do not rely on upfront assumptions and can reveal new gene-disease relationships. Currently, gene-centered design is convenient in therapy development. Taking chromosome 19 as an example, its high alteration may be linked to weight loss based on our results. Furthermore, the weight loss score pinpoints *TP53* and chromosome 19 alterations, which are related to genome doubling [30]. In total, exploratory analyses have suggested that genetic events in cancer may be coupled to the epigenome, where 16 coding mutant genes are needed to further validated (Fig. 2C).

We identify biomarkers that can be applied to skeletal muscle and focus on the targeting and phenotypic throughput data. To transfer information from skeletal muscle to cancer tissues, features selected based on intraclass correlations are subsequently used for prediction in the cancer context. Overall, insights regarding weight loss and skeletal muscle would be conserved after being validated by European cohorts.

The main weaknesses are that the data primarily involve individuals of European descent and lack a fully healthy control group. We do not address the inherent flaws in the data, rather another contribution of the manuscript is the co-methylated blocks (**Table S4**). To determine highly selective oncogenes, restricting co-methylated blocks to the interior of the amplified region may facilitate subsequent functional validation [25].

In conclusion, weight loss can have profound effects on DNAm, which in turn can influence genetics. DNAm data can be combined with experiments to obtain highly promising candidate targets, and also serve as an intermediate medium for establishing new gene-disease relationships.

## Limitations

The study’s conclusions relied on correlation analyses rather than establishing causal effects. For DNA methylation patterns, complementary validation in independent datasets or functional assays could facilitate clinic translatability. In addition, it is desirable to clarify whether four key genes affect weight in the cancer context.

## Electronic supplementary material

Below is the link to the electronic supplementary material.

Supplementary Material 1

Supplementary Material 2

Supplementary Material 3

## Data Availability

This manuscript did not generate new data. Processed data included CPTAC (https://pdc.cancer.gov/pdc/cptac-pancancer), GSE135063, GSE272137, GSE213478, and GSE151407 (https://www.ncbi.nlm.nih.gov/geo/query/acc.cgi? acc=GSE135063; GSE272137; GSE213478; GSE151407). The IMPC (https://www.mousephenotype.org/) and TCDA (http://fcgportal.org/TCDA/) portals were further queried. Meanwhile, part raw data were provided in the supplementary documents.

## Abbreviations

DNAm: DNA methylation
SCNAs: Somatic copy number alterations
CPTAC: Clinical Proteomic Tumor Analysis Consortium
LSCC: Lung squamous carcinoma
LUAD: Lung adenocarcinoma
HNSCC: Head and neck squamous carcinoma
ccRCC: Clear cell renal cell carcinoma
PDAC: Pancreatic ductal adenocarcinoma
BMI: Body mass index
TMB: Tumor mutation burden
CIN: Chromosome instability
GTEx: Genotype-Tissue Expression
