# Supplementary material for: Weight-loss associated DNA methylation patterns: targetable biomarkers and pathway insights
Source: BMC Res Notes. 2025 Jul 22;18:314. doi: 10.1186/s13104-025-07324-x (PMC12281797; doi:10.1186/s13104-025-07324-x)
Supplement: Supplementary file 1 — Supplementary Material 1 [file 13104_2025_7324_MOESM1_ESM.zip › EnMCB/inst/doc/vignette.html]

EnMCB


# EnMCB

#### Xin Yu

#### 2024-04-30

- Introduction
- Installation
- Useage
- Session Info
- References

## Introduction

This package is designed to help you to create the methylation correlated blocks using methylation profiles. A stacked ensemble of machine learning models, which combined the Cox regression, support vector regression and elastic-net regression model, can be constructed using this package1. You also can choose one of them to build DNA methylation signatures associated with disease progression.

Note: This package is still under developing. Some of the functions may change.

Followings are brief insturctions for using this package:

You can install and test our package by downloading source package.

## Installation

```
#if(!requireNamespace("BiocManager", quietly = TRUE))
#    install.packages("BiocManager")
#BiocManager::install("EnMCB")
```

## Useage

First, you need a methylation data set, currently only most common platform ‘Illumina Infinium Human Methylation 450K’ is supported.

You can use your own datasets,or use our demo data.

You can automatically run following:

```
suppressPackageStartupMessages(library(EnMCB))

methylation_dataset<-create_demo()

res<-IdentifyMCB(methylation_dataset)
```

IdentfyMCB() function will calculated Pearson correlation coefficients between the any two CpGs. A value of Pearson correlation coefficients which under the threshold was used to identify boundaries between any two adjacent markers indicating uncorrelated methylation. Markers not separated by a boundary were combined into the MCB. You can extract the MCB information with,

```
MCB<-res$MCBinformation
```

and select some of MCBs for further modeling.

```
MCB<-MCB[MCB[,"CpGs_num"]>=5,]
```

In order to get differentially methylated blocks, one may run following:

```
#simulation for the group data
groups = c(rep("control",200),rep("dis",255))

DiffMCB_resutls<-DiffMCB(methylation_dataset,
                         groups,
                         MCB)$tab
```

In order to build survival models, one may run following:

```
# sample the dataset into training set and testing set
trainingset<-colnames(methylation_dataset) %in% sample(colnames(methylation_dataset),0.6*length(colnames(methylation_dataset)))

testingset<-!trainingset

#build the models
library(survival)
data(demo_survival_data)

models<-metricMCB(MCB,
                    training_set = methylation_dataset[,trainingset],
                    Surv = demo_survival_data[trainingset],
                    Method = "cox",ci = TRUE)

#select the best
onemodel<-models$best_cox_model$cox_model
```

Then, you can predict the risk by the model you build:

```
newcgdata<-data.frame(t(methylation_dataset[,testingset]))
           
prediction_results<-predict(onemodel, newcgdata)
```

In order to build ensemble model, one may run following:

```
# You can choose one of MCBs:
select_single_one=1

em<-ensemble_model(t(MCB[select_single_one,]),
                    training_set=methylation_dataset[,trainingset],
                    Surv_training=demo_survival_data[trainingset])
```

Note that this function only can be used for single MCB only, otherwise the precessing time could be very long.

Then, you can predict the risk by the model you build:

```
em_prediction_results<-ensemble_prediction(ensemble_model = em,
                    prediction_data = methylation_dataset[,testingset])
```

This function will return the single vector with risk scores predicted by ensemble model.

For detailed information, you can find at our references.

## Session Info

```
sessionInfo()
```

```
## R version 4.4.0 beta (2024-04-15 r86425)
## Platform: x86_64-pc-linux-gnu
## Running under: Ubuntu 22.04.4 LTS
## 
## Matrix products: default
## BLAS:   /home/biocbuild/bbs-3.19-bioc/R/lib/libRblas.so 
## LAPACK: /usr/lib/x86_64-linux-gnu/lapack/liblapack.so.3.10.0
## 
## locale:
##  [1] LC_CTYPE=en_US.UTF-8       LC_NUMERIC=C              
##  [3] LC_TIME=en_US.UTF-8        LC_COLLATE=en_US.UTF-8    
##  [5] LC_MONETARY=en_US.UTF-8    LC_MESSAGES=en_US.UTF-8   
##  [7] LC_PAPER=en_US.UTF-8       LC_NAME=C                 
##  [9] LC_ADDRESS=C               LC_TELEPHONE=C            
## [11] LC_MEASUREMENT=en_US.UTF-8 LC_IDENTIFICATION=C       
## 
## time zone: America/New_York
## tzcode source: system (glibc)
## 
## attached base packages:
## [1] stats     graphics  grDevices utils     datasets  methods   base     
## 
## other attached packages:
## [1] survival_3.6-4 EnMCB_1.16.0  
## 
## loaded via a namespace (and not attached):
##  [1] tidyselect_1.2.1     libcoin_1.0-10       dplyr_1.1.4         
##  [4] blob_1.2.4           filelock_1.0.3       fastmap_1.1.1       
##  [7] TH.data_1.1-2        BiocFileCache_2.12.0 pracma_2.4.4        
## [10] digest_0.6.35        rpart_4.1.23         lifecycle_1.0.4     
## [13] cluster_2.1.6        RSQLite_2.3.6        magrittr_2.0.3      
## [16] compiler_4.4.0       rlang_1.1.3          Hmisc_5.1-2         
## [19] sass_0.4.9           tools_4.4.0          partykit_1.2-20     
## [22] utf8_1.2.4           yaml_2.3.8           data.table_1.15.4   
## [25] knitr_1.46           htmlwidgets_1.6.4    bit_4.0.5           
## [28] curl_5.2.1           multcomp_1.4-25      polspline_1.1.24    
## [31] foreign_0.8-86       nnet_7.3-19          grid_4.4.0          
## [34] fansi_1.0.6          colorspace_2.1-0     ggplot2_3.5.1       
## [37] MASS_7.3-60.2        scales_1.3.0         iterators_1.0.14    
## [40] cli_3.6.2            mvtnorm_1.2-4        inum_1.0-5          
## [43] rmarkdown_2.26       rms_6.8-0            generics_0.1.3      
## [46] rstudioapi_0.16.0    httr_1.4.7           DBI_1.2.2           
## [49] cachem_1.0.8         stringr_1.5.1        splines_4.4.0       
## [52] nnls_1.5             parallel_4.4.0       base64enc_0.1-3     
## [55] vctrs_0.6.5          sandwich_3.1-0       boot_1.3-30         
## [58] glmnet_4.1-8         Matrix_1.7-0         SparseM_1.81        
## [61] jsonlite_1.8.8       survivalsvm_0.0.5    bit64_4.0.5         
## [64] Formula_1.2-5        htmlTable_2.4.2      foreach_1.5.2       
## [67] jquerylib_0.1.4      glue_1.7.0           stabs_0.6-4         
## [70] codetools_0.2-20     mboost_2.9-10        stringi_1.8.3       
## [73] shape_1.4.6.1        gtable_0.3.5         quadprog_1.5-8      
## [76] munsell_0.5.1        tibble_3.2.1         pillar_1.9.0        
## [79] quantreg_5.97        htmltools_0.5.8.1    R6_2.5.1            
## [82] dbplyr_2.5.0         survivalROC_1.0.3.1  evaluate_0.23       
## [85] lattice_0.22-6       backports_1.4.1      memoise_2.0.1       
## [88] bslib_0.7.0          MatrixModels_0.5-3   Rcpp_1.0.12         
## [91] nlme_3.1-164         gridExtra_2.3        checkmate_2.3.1     
## [94] xfun_0.43            zoo_1.8-12           pkgconfig_2.0.3
```

## References

---

1. Xin Yu et al. 2019 Predicting disease progression in lung adenocarcinoma patients based on methylation correlated blocks using ensemble machine learning classifiers (under review)↩︎
