# Supplementary material for: Weight-loss associated DNA methylation patterns: targetable biomarkers and pathway insights
Source: BMC Res Notes. 2025 Jul 22;18:314. doi: 10.1186/s13104-025-07324-x (PMC12281797; doi:10.1186/s13104-025-07324-x)
Supplement: Supplementary file 2 — Supplementary Material 2 [file 13104_2025_7324_MOESM2_ESM.docx]

**SUPPLEMENTARY INFORMATION**


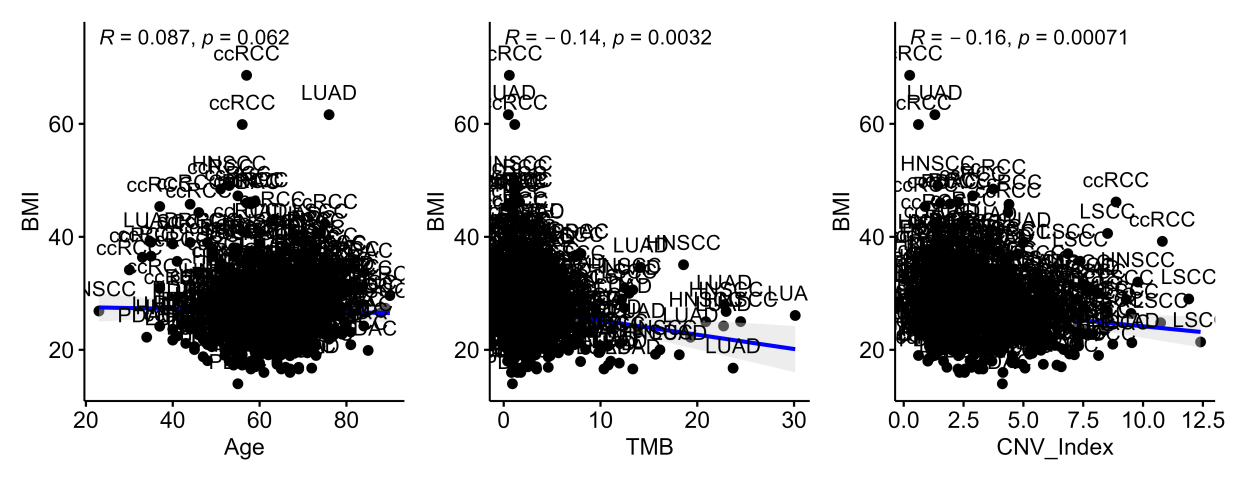


**Figure S1**. Inter-class correlation analysis of BMI, age, tumor mutational load and chromosome instability scores in five types CPTAC tumor tissues. Focusing on the potential link to BMI.


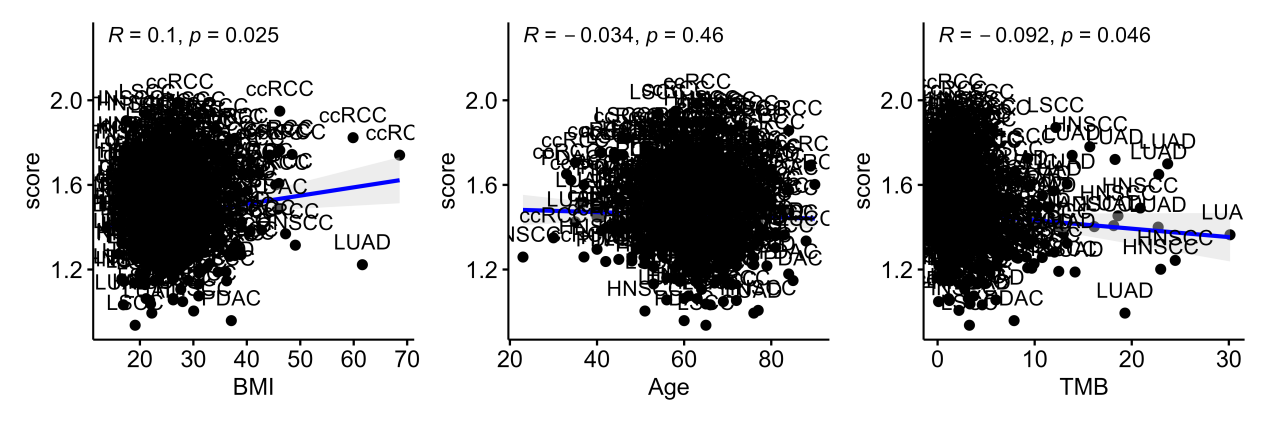


**Figure S2**. Inter-class correlation analysis of DNAm score and BMI, age, and tumor mutational load in five types CPTAC tumor tissues. Focusing on the potential link to DNAm score.


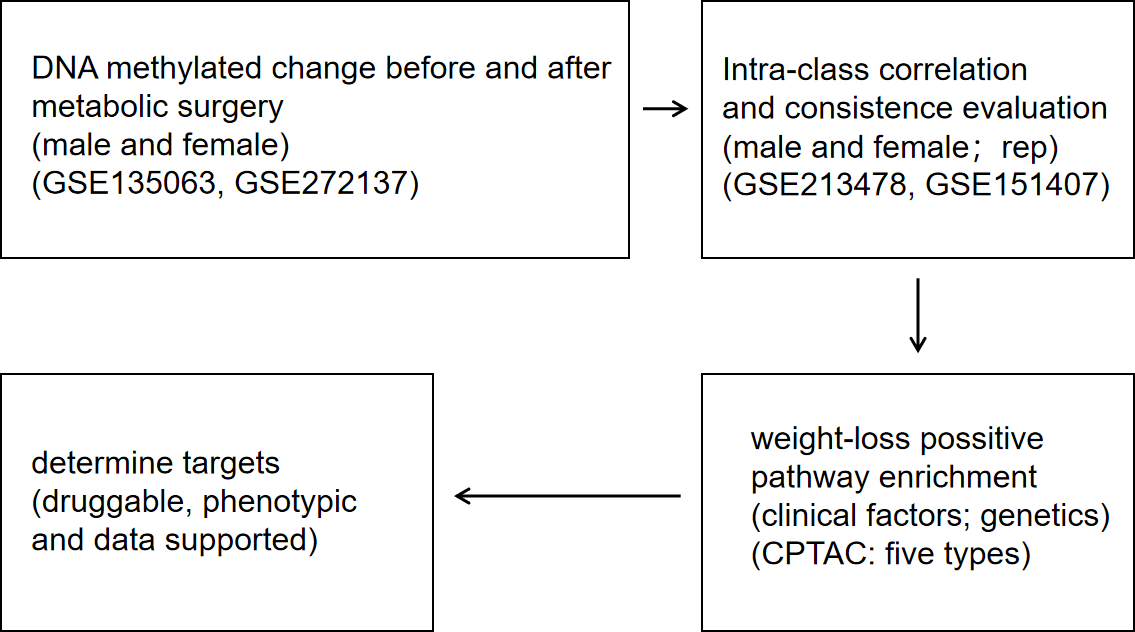


**Figure S3**. Workflow charts used in this article. The charts provide a visual summary of approaches, including the selection of datasets and the information of analytical steps performed.
